# Prognostic utility of sestamibi lung uptake does not require adjustment for stress-related variables: A retrospective cohort study

**DOI:** 10.1186/1471-2385-6-2

**Published:** 2006-03-29

**Authors:** William D Leslie, Marina S Yogendran, Linda M Ward, Khaled A Nour, Colleen J Metge

**Affiliations:** 1 Department of Medicine (C5121) 409 Tache Avenue, Winnipeg, R2H 2A6, Canada

## Abstract

**Background:**

Increased ^99m^Tc-sestamibi stress lung-to-heart ratio (sLHR) has been shown to predict cardiac outcomes similar to pulmonary uptake of thallium. Peak heart rate and use of pharmacologic stress affect the interpretation of lung thallium uptake. The current study was performed to determine whether ^99m^Tc-sestamibi sLHR measurements are affected by stress-related variables, and whether this in turn affects prognostic utility.

**Methods:**

sLHR was determined in 718 patients undergoing ^99m^Tc-sestamibi SPECT stress imaging. sLHR was assessed in relation to demographics, hemodynamic variables and outcomes (mean follow up 5.6 ± 1.1 years).

**Results:**

Mean sLHR was slightly greater in males than in females (P < 0.01) and also showed a weak negative correlation with age (P < 0.01) and systolic blood pressure (P < 0.01), but was unrelated to stress method or heart rate at the time of injection. In patients undergoing treadmill exercise, sLHR was also positively correlated with peak workload (P < 0.05) but inversely with double product (P < 0.05). The combined explanatory effect of sex, age and hemodynamic variables on sLHR was less than 10%. The risk of acute myocardial infarction (AMI) or death increased by a factor of 1.7–1.8 for each SD increase in unadjusted sLHR, and was unaffected by adjustment for sex, age and hemodynamic variables (hazard ratios 1.6–1.7). The area under the ROC curve for the unadjusted sLHR was 0.65 (95% CI 0.59–0.71, P < 0.0001) and was unchanged for the adjusted sLHR (0.65, 95% CI 0.61–0.72, P < 0.0001).

**Conclusion:**

Stress-related variables have only a weak effect on measured sLHR. Unadjusted and adjusted sLHR provide equivalent prognostic information for prediction of AMI or death.

## Background

The assessment of myocardial perfusion using radioactive tracers has consistently been shown to provide important prognostic information in patients with known or suspected coronary artery disease [1-3]. A number of additional non-perfusion parameters of left ventricular function have also been described to have prognostic importance, such as transient ischemic cavity dilation (TID) and left ventricular systolic function [4,5]. Increased post-stress ^99m^Tc-sestamibi lung-to-heart ratio (sLHR) has recently been shown to predict cardiac outcomes independent of other clinical and imaging variables [6]. Peak heart rate and use of pharmacologic stress affect post-stress pulmonary uptake of thallium-201, and these factors need to be considered when interpreting lung thallium measurements [7-9]. Lung activity on exercise thallium studies is inversely related to peak heart rate and propranolol use, and the use of adjusted reference ranges has been advocated [7]. It is currently unknown how stress-related variables influence ^99m^Tc-sestamibi sLHR and whether this in turn affects its prognostic utility and clinical interpretation.

## Methods

### Study population

Between January 1994 and April 1999, consecutive patients who underwent stress and rest ^99m^Tc-sestamibi SPECT myocardial perfusion imaging on the same imaging system (Elscint 409; Haifa, Israel) were considered for inclusion in the study cohort (n = 1,027). The study cohort, stress procedures and image processing used in our laboratory have been previously described [10]. Exclusion criteria included planar imaging or imaging not completed on the designated camera (n = 36), corrupted storage media (n = 102) or poor quality image data (n = 2), non-standard stress procedures (n = 4), or inability to link patient data to the Manitoba Population Health Research Data Repository (n = 140). For patients undergoing more than one scan during the study period, only the first scan was included in the analysis (n = 25). The study protocol was approved by local Research Ethics Board and provincial Health Information Privacy Committee of Manitoba Health. Individual patient consent was not required for this retrospective study in compliance with local legislation governing the use of personal health information.

### Stress protocols and imaging

A two-day protocol utilizing treadmill exercise is the preferred procedure in our laboratory. A one-day (rest followed by stress) procedure was used in a small number of cases (39 [5 %]). Whenever possible, beta blockers and calcium-channel antagonists are withheld for 24–48 hours prior to the stress procedure, and nitrates are avoided for at least six hours. Symptom-limited treadmill exercise is performed with tracer injection at peak exercise followed by 1–3 minutes of exercise post-injection. For individuals unable to achieve a satisfactory exercise workload, pharmacologic stress with dipyridamole 0.56 mg/kg is administered intravenously over 4 minutes with tracer injection 4 1/2 minutes later. Low-level (supplementary) exercise is performed following dipyridamole infusion for those patients without a left bundle branch block whenever possible. Supine, non-gated SPECT imaging is commenced 30–60 minutes post-stress and 45–75 minutes following the resting tracer injections.

### Scintigraphic interpretation

#### Visual and quantitative perfusion analysis

An initial visual interpretation of the scan data was performed without image quantification by a pair of nuclear medicine specialists with extensive experience in cardiac nuclear medicine. Images were categorized as *normal*, *equivocal*, *abnormal with fixed defects*, *abnormal with fully reversible defects*, or *abnormal with partially reversible defects*. For analytic purposes, these categories were recoded using two variables as follows: normal scan (normal or equivocal) versus abnormal scan (abnormal with fixed or reversible defects); no reversibility (normal, equivocal or abnormal with fixed defects) versus reversibility (abnormal with fully reversible or partially reversible defects). The image data was subsequently reprocessed using commercially available software (AutoSPECT and QPS AutoQUANT, Cedar Sinai Medical Centre and ADAC Laboratories, Milpitas, CA) [11-13]. Left ventricular contours were checked visually and manually adjusted if the computer-generated automatic contours were found to be incorrect. Briefly, this software provides a quantitative defect extent and severity measurement defined from gender-specific normal limits by adding the scores from twenty left ventricular segments (0 = normal to 4 = absent uptake) on the stress sestamibi images, and is called the summed stress score (SSS) [14,15]. The summed rest score (SRS) and summed difference score (SDS) reflect resting perfusion abnormalities and the change between the stress and rest perfusion scores, respectively. We have previously shown that there is close agreement between the visual and automated quantitative assessment in terms of diagnosis and prognosis [10,16]. An automated calculation of ungated left ventricular volumes post-stress and at rest with their ratio (referred to as transient ischemic dilation [TID]) was obtained [17]. Visual interpretation and the quantitative analysis were performed without knowledge of patient outcomes.

#### Lung uptake measurements

Lung uptake of ^99m^Tc-sestamibi was calculated as a stress lung-to-heart ratio (sLHR) using an automated technique previously validated by Bacher-Stier et al [18]. The algorithm sums five adjacent projection images centered on the anterior view, and then automatically identifies myocardial borders and a crescentic lung region. Lung uptake is then expressed as the ratio of average lung uptake per pixel divided by maximum heart uptake per pixel in a 4 × 4 pixel region containing the hottest cardiac pixel. Interobserver variation in LHR measurements was assessed in 30 cases that were completely re-processed by an individual not involved in the original analysis and who was blinded to all clinical information and the previous results. There was excellent agreement in the sLHR (R = 0.97, P < 0.00001).

### Outcomes assessment

Cohort follow up was performed through the Manitoba Population Health Research Data Repository which contains anonymized encounter-based records of individuals' interactions with the provincial health care system, including physician services and hospitalizations for all residents of the province of Manitoba [19]. These administrative data have been validated and used in a wide range of clinical disorders, including prediction of mortality following acute myocardial infarction (AMI) [20-22].

The primary outcome of this study was death or AMI. The date of death was established from the Vital Statistics database. AMI was defined from hospital discharge diagnosis (ICD-9-CM 410.xx). Clinical and stress variables were primarily obtained from chart review. The data repository was used to identify individuals with diabetes or previous hospitalization with AMI using validated definitions [22,23].

### Statistical analysis

Statistical analysis was performed with a commercial software package (Statistica Version 6.1, StatSoft Inc, Tulsa, OK). Continuous variables are reported as mean ± SD and P < 0.05 is considered to represent a statistically significant difference. Group comparisons were performed using ANOVA (continuous variables) or a Chi-square test (categorical variables). Logistic regression was used to identify the independent correlates of an elevated sLHR (defined as the 95% upper confidence interval for event-free patients with visually normal perfusion and SSS ≤ 3). Cox proportional hazards model was used to estimate relative event rates from sLHR before and after adjustment for stress-related correlates. Receiver operating curve (ROC) analysis was used to assess overall risk stratification for single multi-level variables (SPSS Version 11.0, SPSS Inc, Chicago, IL).

## Results

The selection criteria identified 1,027 potentially eligible cases. Exclusion criteria were present in 309 of these leaving a final study population of 718 patients. Excluded patients were similar to the final study cohort in terms of age, gender, stress procedure and frequency of abnormal scans. The mean age of the cohort was 60 ± 11 years. Of the 718 patients included in the analysis, 380 (53%) were male, 186 (26%) had a history of MI, 112 (16%) had undergone prior coronary bypass surgery or coronary angioplasty, 90 (13%) were diabetic, and 556 (77%) had treadmill exercise as the only stress method. As expected, heart rate at the time of tracer injection was greater with exercise stress alone (131 ± 21 BPM) than for dipyridamole alone (87 ± 20 BPM) or dipyridamole combined with supplemental exercise (103 ± 19 BPM) (P < 0.0001). Similarly, systolic blood pressure varied according to stress method (exercise only 171 ± 27 mmHg, dipyridamole only 134 ± 29 mmHg, dipyridamole combined with supplemental exercise 143 ± 22 mmHg; P < 0.0001).

sLHR was positively correlated with SSS (R = 0.38, P < 0.0001), though SSS explained less than 15% of the variance in sLHR. Of the 718 patients, 69 (10%) had sLHR values that exceeded the normal range (≤ 0.39). Factors that were associated with elevated sLHR are summarized in Table 1 and include male sex, previous heart ischemic disease (AMI or revascularization), diabetes, greater perfusion abnormalities, and larger left ventricular cavity measurements. Logistic regression showed that only SSS (P = 0.0003), stress left ventricular volume (P < 0.0001) and previous revascularization (P = 0.04) were independent predictors of elevated sLHR.

**Table 1:** Cohort characteristics, stress procedure and scan findings in relation to elevated stress lung heart ratio (sLHR).

	Normal sLHR (n = 649)	Elevated sLHR (n = 69)	P
**Clinical variables**			
Age (years)	61 ± 11	59 ± 12	>0.2
Sex (male)	325 (50%)	55 (80%)	<0.0001
Previous AMI	154 (24%)	32 (46%)	<0.0001
Previous revascularization	93 (14%)	19 (28%)	0.004
Diabetes	74 (11%)	16 (23%)	0.005
**Stress Modality**			
Exercise only	505 (78%)	51 (74%)	>0.2
Dipyridimole ^a^	144 (22%)	18 (26%)	>0.2
**Perfusion**			
SSS	6 ± 8	16 ± 12	<0.0001
SRS	2 ± 5	8 ± 9	<0.0001
SDS	3 ± 4	7 ± 5	<0.0001
**LV Volume**			
Stress (ml)	72 ± 43	141 ± 100	<0.0001
Rest (ml)	70 ± 40	133 ± 96	<0.0001
TID	1.02 ± 0.13	1.06 ± 0.16	0.02

^a ^dipyridamole alone or combined with supplemental exercise

Clinical and hemodynamic variables that might affect interpretation of lung uptake measurements were assessed in the 334 event-free patients with normal perfusion (visually normal and SSS = 3) (Table 2). Of these, 123 (37%) were male and 284 (85%) had treadmill exercise as the only stress method with the remainder receiving either dipyridamole alone (32 [10%]) or combined with supplemental exercise (18 [5%]). Mean sLHR was slightly greater in males than in females (0.31 ± 0.05 vs. 0.30 ± 0.05, P < 0.01) and also showed a weak negative correlation with age (R = -0.17, P < 0.01). There was an inverse correlation with systolic blood pressure (R = -0.11, P < 0.01), but no relationship with heart rate at the time of injection (R = -0.01, P > 0.2). Mean sLHR was identical for individuals undergoing exercise and dipyridamole stress (0.30 ± 0.05 vs. 0.30 ± 0.04, P > 0.2). In the multivariable regression analysis (Table 3) sex, age and systolic blood pressure remained independently associated with sLHR but the combined explanatory effect was weak (7.5% of total variance).

**Table 2:** Univariate correlates with stress lung-heart-ratios (sLHR). Hemodynamic measurements were recorded at the time of tracer injection. Continuous variables were stratified according to the median.

	Combined stress methods (n = 334)		Exercise stress only (n = 284)	
	Mean sLHR	R value	Mean sLHR	R value

Sex				
Male	0.31 ± 0.05^b^	-0.16^b^	0.31 ± 0.05^b^	-0.16^b^
Female	0.30 ± 0.05		0.30 ± 0.05	
Age				
> median (58 years)	0.30 ± 0.05 ^a^	-0.17^b^	0.30 ± 0.05^a^	-0.19^c^
< median	0.31 ± 0.05		0.31 ± 0.05	
Heart rate				
> median (135 BPM)	0.30 ± 0.05	-0.01	0.30 ± 0.05	-0.05
< median	0.30 ± 0.05		0.30 ± 0.05	
Systolic BP				
> median (175 mmHg)	0.30 ± 0.05^b^	-0.11^a^	0.30 ± 0.05^b^	-0.15^a^
< median	0.31 ± 0.05		0.31 ± 0.05	
Diastolic BP				
> median (85 mmHg)	0.30 ± 0.05	-0.06	0.31 ± 0.05	-0.09
< median	0.30 ± 0.05		0.30 ± 0.05	
Stress method				
Exercise only	0.30 ± 0.05	0.00	--	--
Dipyridamole^d^	0.30 ± 0.04		--	
Peak workload				
> median (8.7 METS)	--	--	0.31 ± 0.05^a^	0.15 ^a^
< median	--		0.30 ± 0.05	
Double product (SBP × HR)				
> median (24,000)	--	--	0.30 ± 0.05^a^	-0.12^a^
< median	--		0.31 ± 0.05	

^a ^P < 0.05, ^b ^P < 0.01, ^c ^P < 0.001, ^d ^dipyridamole alone or combined with supplemental exercise

**Table 3:** Multivariate regression of correlates with stress lung-heart-ratios (sLHR).

	Combined stress methods (n = 334)	Exercise stress only (n = 284)
Factor (P value)	Sex (0.0025)	Sex (0.0041)
	Age (0.0058)	Age (0.0002)
	Systolic blood pressure (0.0068)	Peak double product (0.0017)
Global R^2 ^(P value)	0.075 (<0.0001)	0.083 (<0.0001)

In the 284 event-free patients with normal perfusion undergoing treadmill exercise only, sLHR was again unrelated to peak heart rate (R = -0.05, P > 0.2) but was positively correlated with peak workload (R = 0.15, P < 0.05) and inversely related to systolic blood pressure (R = -0.15, P < 0.01) and double product (R = -0.12, P < 0.05). Peak double product was used in the multivariable regression model since it explained slightly more of the variance in sLHR than systolic blood pressure. Peak workload was no longer a significant variable after adjustment for the other variables.

**Figure 1:**
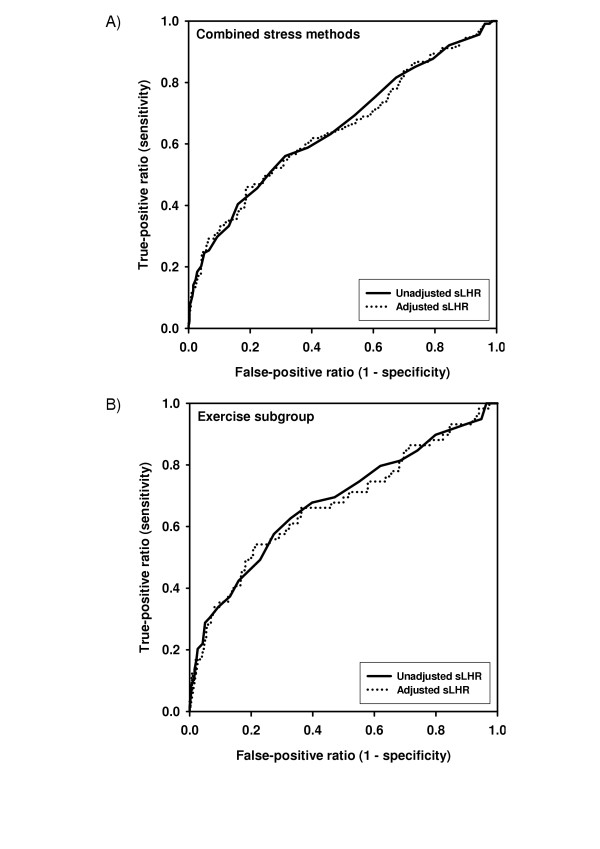
**Receiver operating characteristic (ROC) curves for prediction of acute myocardial infarction or death**. ROC curves for unadjusted (solid line) and adjusted (dotted line) stress lung-heart-ratios (sLHR) are plotted in relation to adverse outcomes. (A) Combined stress methods (adjusted for sex, age and systolic blood pressure). (B) Subgroup of patients undergoing treadmill exercise only (adjusted for sex, age and peak double product).

The mean period of follow up was 5.6 ± 1.1 years. During this time, 114 (16%) from the cohort experienced a primary outcome event. There were 81 deaths and 62 acute myocardial infarctions, including 29 patients recorded as having AMI followed by death. For the latter, time to AMI was used in the survival analysis. The hazard ratio (HR) for an adverse event increased by a factor of 1.7 (95% CI 1.4–1.9) for each SD increase in unadjusted sLHR. This was no different than for sLHR after adjustment for sex, age and systolic blood pressure (HR 1.6, 95% CI 1.4–1.9). ROC analysis confirmed a significant association between adverse outcomes over the range of sLHR measurements (Figure 1). The area under the curve for unadjusted sLHR was 0.65 (95% CI 0.59–0.71, P < 0.0001 versus null hypothesis of area = 0.5) which is identical to the area under the curve for sLHR adjusted for sex, age and systolic blood pressure (0.65, 95% CI 0.59–0.71, P < 0.0001).

Among exercise-only subjects with normal perfusion, there was no significant improvement in risk stratification following adjustment for sex, age, and peak double-product (area under ROC curve for unadjusted sLHR 0.68, 95% CI 0.60–0.76, P < 0.0001 vs. adjusted sLHR 0.67, 95% CI 0.59–0.75, P < 0.0001). The relative hazard for an adverse event increased by a factor of 1.8 (95% CI 1.5–2.1) for each SD increase in unadjusted sLHR, similar to the adjusted sLHR (HR 1.7, 95% CI 1.4–2.0).

## Discussion

This report shows that sex, age and hemodynamic variables have only a very weak effect on ^99m^Tc-sestamibi stress lung uptake and together these variables explain less than 10% of variation in the sLHR. The risk of acute myocardial infarction (AMI) or death increased by a factor of 1.7–1.8 for each SD increase in unadjusted sLHR, and this was similar after adjustment for sex, age and hemodynamic variables (hazard ratios 1.6–1.7). The area under the ROC curve for the unadjusted sLHR was also similar to the adjusted sLHR. It has previously been shown that the sLHR gives incremental predictive information when added to a model containing clinical, stress, perfusion and left ventricular volume information [6]. The current study indicates that interpretation of the sLHR is not appreciably affected by stress-related variables, which contrasts with lung thallium uptake.

Thallium lung uptake has been shown to be affected by hemodynamic variables and imaging time. In 59 normal patients, lower peak heart rate during exercise and propranolol peak heart rate during exercise and propranolol usage were both associated with higher lung uptake [7] and this has been confirmed in subsequent studies [24]. The use of a normal range for lung thallium uptake that is adjusted for peak heart rate has been proposed but has not been widely adopted [7]. In contrast, we were unable to find any relationship between heart rate at the time of sestamibi injection and sLHR. The difference in tracer characteristics may in part relate to the transient nature of lung thallium uptake best detected when imaging begins within minutes of exercise [9]. Our protocol for sestamibi imaging does not occur until 30–60 minutes following the stress injection. In conjunction with the slower rate of sestamibi extraction, this may attenuate the effect of heart rate. sLHR did show a modest inverse correlation with peak double product in those subjects undergoing only treadmill exercise.

We found slightly higher sLHR in males with normal perfusion scans than in females and this has also been reported by others [25]. This gender effect cannot be explained from differences in peak heart rate, but may reflect differences in chest wall tissue composition and attenuation. Women have been shown to have significantly higher resting and hyperemic myocardial blood flow than men, perhaps related to differences in lipid profiles or estrogen [26-28]. Alternatively, gender may simply be a marker for other factors associated with elevated sLHR since it was not a significant independent predictor in the multivariate adjusted model. The reason for the correlation with age is also uncertain, but a significant reduction in hyperemic flow has been reported after age 70 [29]. The net effect of age and gender was quite weak and explained less than 5% of the variance in sLHR, and less than 10% if systolic blood pressure or peak double product were also considered. Analysis based upon age-, gender- and stress-adjusted sLHR did not affect the survival analysis. Therefore, we do not believe that adjusted normal ranges are required. Finally, we did not find any difference in sLHR according to whether the patients performed exercise only or underwent pharmacologic stress. Once again, this may relate to the relatively long delay between the stress procedure and subsequent imaging when compared with thallium stress imaging protocols. It suggests that the same normal range can be used independent of the stress modality.

A limitation of our study is the absence of the additional functional information that comes from gated SPECT imaging. Gated SPECT is performed in most nuclear cardiology laboratories and provides an assessment of regional and global left ventricular function. Left ventricular ejection fraction derived from gated SPECT myocardial perfusion imaging contributes independent prognostic information [30].

## Conclusion

sLHR appears to be another adjunctive prognostic measure in patients with known or suspected coronary artery disease. Age, gender and stress variables show only weak correlations with sLHR, and adjustment for these factors does not appear to be necessary for interpreting sLHR measurements. These and other variables, such as regional perfusion and systolic function, remain important prognostic variables which must be considered in patient risk stratification.

## Competing interests

The author(s) declare that they have no competing interests.

## Authors' contributions

WDL developed the study protocol, performed the data analysis and drafted the manuscript

MSY performed the data linkage and outcomes data extraction

LMW restored the archived scan data, performed the QPS re-analysis and assembled the clinical datasets

KAN contributed to the study protocol and data interpretation

CJM obtained approvals from Manitoba Health and the Manitoba Centre for Health Policy, and coordinated anonymization of the clinical information

All authors read and approved the final manuscript.

## Pre-publication history

The pre-publication history for this paper can be accessed here:



## References

[B1] Berman DS, Hachamovitch R, Kiat H, Cohen I, Cabico JA, Wang FP, Friedman JD, Germano G, Van Train K, Diamond GA (1995). Incremental value of prognostic testing in patients with known or suspected ischemic heart disease: a basis for optimal utilization of exercise technetium-99m sestamibi myocardial perfusion single-photon emission computed tomography. J Am Coll Cardiol.

[B2] Hachamovitch R, Berman DS, Kiat H, Cohen I, Cabico JA, Friedman J, Diamond GA (1996). Exercise myocardial perfusion SPECT in patients without known coronary artery disease: incremental prognostic value and use in risk stratification. Circulation.

[B3] Hachamovitch R, Berman DS, Shaw LJ, Kiat H, Cohen I, Cabico JA, Friedman J, Diamond GA (1998). Incremental prognostic value of myocardial perfusion single photon emission computed tomography for the prediction of cardiac death: differential stratification for risk of cardiac death and myocardial infarction. Circulation.

[B4] Hansen CL, Sangrigoli R, Nkadi E, Kramer M (1999). Comparison of pulmonary uptake with transient cavity dilation after exercise thallium-201 perfusion imaging. J Am Coll Cardiol.

[B5] Klocke FJ, Baird MG, Lorell BH, Bateman TM, Messer JV, Berman DS, O'Gara PT, Carabello BA, Russell ROJ, Cerqueira MD, John Sutton MG, DeMaria AN, Udelson JE, Kennedy JW, Verani MS, Williams KA, Antman EM, Smith SCJ, Alpert JS, Gregoratos G, Anderson JL, Hiratzka LF, Faxon DP, Hunt SA, Fuster V, Jacobs AK, Gibbons RJ, Russell RO (2003). ACC/AHA/ASNC guidelines for the clinical use of cardiac radionuclide imaging--executive summary: a report of the American College of Cardiology/American Heart Association Task Force on Practice Guidelines (ACC/AHA/ASNC Committee to Revise the 1995 Guidelines for the Clinical Use of Cardiac Radionuclide Imaging). J Am Coll Cardiol.

[B6] Leslie WD, Tully SA, Yogendran MS, Ward LM, Nour KA, Metge CJ (2004). Prognostic value of lung sestamibi uptake in myocardial perfusion imaging of patients with known or suspected coronary artery disease. J Am Coll Cardiol.

[B7] Brown KA, Boucher CA, Okada RD, Strauss HW, Pohost GM (1984). Quantification of pulmonary thallium-201 activity after upright exercise in normal persons: importance of peak heart rate and propranolol usage in defining normal values. Am J Cardiol.

[B8] Nishimura T, Uehara T, Hayashida K, Kozuka T, Saito M, Sumiyoshi T (1987). Quantitative assessment of thallium myocardial washout rate: importance of peak heart rate and lung thallium uptake in defining normal values. Eur J Nucl Med.

[B9] Mahmood S, Buscombe JR, Ell PJ (1992). The use of thallium-201 lung/heart ratios. Eur J Nucl Med.

[B10] Leslie WD, Tully SA, Yogendran MS, Ward LM, Nour KA, Metge CJ (2004). Automated quantification of 99mTc sestamibi myocardial perfusion compared with visual analysis. Nucl Med Commun.

[B11] Germano G, Kavanagh PB, Chen J, Waechter P, Su HT, Kiat H, Berman DS (1995). Operator-less processing of myocardial perfusion SPECT studies. J Nucl Med.

[B12] Germano G, Kavanagh PB, Su HT, Mazzanti M, Kiat H, Hachamovitch R, Van Train KF, Areeda JS, Berman DS (1995). Automatic reorientation of three-dimensional, transaxial myocardial perfusion SPECT images. J Nucl Med.

[B13] Germano G, Kavanagh PB, Berman DS (1997). An automatic approach to the analysis, quantitation and review of perfusion and function from myocardial perfusion SPECT images. Int J Card Imaging.

[B14] Germano G, Kavanagh PB, Waechter P, Areeda J, Van Kriekinge S, Sharir T, Lewin HC, Berman DS (2000). A new algorithm for the quantitation of myocardial perfusion SPECT. I: technical principles and reproducibility. J Nucl Med.

[B15] Sharir T, Germano G, Waechter PB, Kavanagh PB, Areeda JS, Gerlach J, Kang X, Lewin HC, Berman DS (2000). A new algorithm for the quantitation of myocardial perfusion SPECT. II: validation and diagnostic yield. J Nucl Med.

[B16] Leslie WD, Tully SA, Yogendran MS, Ward LM, Nour KA, Metge CJ (2005). Prognostic value of automated quantification of 99mTc-sestamibi myocardial perfusion imaging. J Nucl Med.

[B17] Abidov A, Bax JJ, Hayes SW, Hachamovitch R, Cohen I, Gerlach J, Kang X, Friedman JD, Germano G, Berman DS (2003). Transient ischemic dilation ratio of the left ventricle is a significant predictor of future cardiac events in patients with otherwise normal myocardial perfusion SPECT. J Am Coll Cardiol.

[B18] Bacher-Stier C, Sharir T, Kavanagh PB, Lewin HC, Friedman JD, Miranda R, Germano G, Berman DS (2000). Postexercise lung uptake of 99mTc-sestamibi determined by a new automatic technique: validation and application in detection of severe and extensive coronary artery disease and reduced left ventricular function. J Nucl Med.

[B19] Roos NP, Shapiro E (1999). Revisiting the Manitoba Centre for Health Policy and Evaluation and its population-based health information system. Med Care.

[B20] Roos LL, Mustard CA, Nicol JP, McLerran DF, Malenka DJ, Young TK, Cohen MM (1993). Registries and administrative data: organization and accuracy. Med Care.

[B21] Roos LL, Sharp SM, Wajda A (1989). Assessing data quality: a computerized approach. Soc Sci Med.

[B22] Tu JV, Austin PC, Walld R, Roos L, Agras J, McDonald KM (2001). Development and validation of the Ontario acute myocardial infarction mortality prediction rules. J Am Coll Cardiol.

[B23] Blanchard JF, Ludwig S, Wajda A, Dean H, Anderson K, Kendall O, Depew N (1996). Incidence and prevalence of diabetes in Manitoba, 1986-1991. Diabetes Care.

[B24] Nordrehaug JE, Danielsen R, Vik-Mo H (1990). Physiological inverse relationship between heart rate and thallium-201 lung uptake, clearance and lung/myocardial uptake ratio. Eur Heart J.

[B25] Romanens M, Gradel C, Saner H, Pfisterer M (2001). Comparison of 99mTc-sestamibi lung/heart ratio, transient ischaemic dilation and perfusion defect size for the identification of severe and extensive coronary artery disease. Eur J Nucl Med.

[B26] Berman M, Gewirtz H (1997). Acute effects of 17 beta-estradiol on the coronary microcirculation: observations in sedated, closed-chest domestic swine. Coron Artery Dis.

[B27] Duvernoy CS, Meyer C, Seifert-Klauss V, Dayanikli F, Matsunari I, Rattenhuber J, Hoss C, Graeff H, Schwaiger M (1999). Gender differences in myocardial blood flow dynamics: lipid profile and hemodynamic effects. J Am Coll Cardiol.

[B28] Chareonthaitawee P, Kaufmann PA, Rimoldi O, Camici PG (2001). Heterogeneity of resting and hyperemic myocardial blood flow in healthy humans. Cardiovasc Res.

[B29] Uren NG, Camici PG, Melin JA, Bol A, de Bruyne B, Radvan J, Olivotto I, Rosen SD, Impallomeni M, Wijns W (1995). Effect of aging on myocardial perfusion reserve. J Nucl Med.

[B30] Sharir T, Germano G, Kavanagh PB, Lai S, Cohen I, Lewin HC, Friedman JD, Zellweger MJ, Berman DS (1999). Incremental prognostic value of post-stress left ventricular ejection fraction and volume by gated myocardial perfusion single photon emission computed tomography. Circulation.

